# Distance-related functional reorganization predicts motor outcome in stroke patients

**DOI:** 10.1186/s12916-024-03435-7

**Published:** 2024-06-18

**Authors:** Wenjun Hong, Zaixing Liu, Xin Zhang, Ming Li, Zhixuan Yu, Yuxin Wang, Minmin Wang, Yanan Wu, Shengjie Fang, Bo Yang, Rong Xu, Zhiyong Zhao

**Affiliations:** 1 Department of Rehabilitation Medicine, Nanjing Drum Tower Hospital, Affiliated Hospital of Medical School, Nanjing University, Nanjing, 210008 China; 2 Department of Radiology, Nanjing Drum Tower Hospital, Affiliated Hospital of Medical School, Nanjing University, Nanjing, 210008 China; 3 School of Biomedical Engineering and Instrument Science, Zhejiang University, Hangzhou, 310027 China; 4 Binjiang Institute of Zhejiang University, Hangzhou, 310014 China; 5 Department of Rehabilitation Medicine, Nanjing Drum Tower Hospital Clinical College of Jiangsu University, Nanjing, 210008 China; 6 Department of Radiology, Children’s Hospital, Zhejiang University School of Medicine, National Clinical Research Center for Child Health, Hangzhou, 310003 China

**Keywords:** Stroke, Distance, Functional connectivity density, Motor function, Resting-state fMRI, Biomarker

## Abstract

**Background:**

Analyzing distance-dependent functional connectivity density (FCD) yields valuable insights into patterns of brain activity. Nevertheless, whether alterations of FCD in non-acute stroke patients are associated with the anatomical distance between brain regions remains unclear. This study aimed to explore the distance-related functional reorganization in non-acute stroke patients following left and right hemisphere subcortical lesions, and its relationship with clinical assessments.

**Methods:**

In this study, we used resting-state fMRI to calculate distance-dependent (i.e., short- and long-range) FCD in 25 left subcortical stroke (LSS) patients, 22 right subcortical stroke (RSS) patients, and 39 well-matched healthy controls (HCs). Then, we compared FCD differences among the three groups and assessed the correlation between FCD alterations and paralyzed motor function using linear regression analysis.

**Results:**

Our findings demonstrated that the left inferior frontal gyrus displayed distance-independent FCD changes, while the bilateral supplementary motor area, cerebellum, and left middle occipital gyrus exhibited distance-dependent FCD alterations in two patient subgroups compared with HCs. Furthermore, we observed a positive correlation between increased FCD in the bilateral supplementary motor area and the motor function of lower limbs, and a negative correlation between increased FCD in the left inferior frontal gyrus and the motor function of both upper and lower limbs across all stroke patients. These associations were validated by using a longitudinal dataset.

**Conclusions:**

The FCD in the cerebral and cerebellar cortices shows distance-related changes in non-acute stroke patients with motor dysfunction, which may serve as potential biomarkers for predicting motor outcomes after stroke. These findings enhance our comprehension of the neurobiological mechanisms driving non-acute stroke.

**Trial registration:**

All data used in the present study were obtained from a research trial registered with the ClinicalTrials.gov database (NCT05648552, registered 05 December 2022, starting from 01 January 2022).

**Supplementary Information:**

The online version contains supplementary material available at 10.1186/s12916-024-03435-7.

## Background

During the non-acute stroke stage, survivors continue to undergo a dynamic process of functional reorganization associated with post-stroke recovery [1, 2]. Resting-state fMRI has emerged as a promising avenue to explore brain functional integration and separation after stroke [3]. Our prior resting-state fMRI studies found decreased functional connectivity (FC) between hippocampal subfields and left postcentral gyrus as well as right middle occipital gyrus [4], between cerebellum posterior lobe and left precentral gyrus, inferior frontal gyrus as well as middle temporal gyrus [5], and increased FC from the ipsilesional primary motor cortex to the ipsilesional occipital lobes [6] in non-acute stroke patients compared with healthy controls (HCs). However, the FC analysis needs a predefined selection of regions of interest, which is a challenging issue in neuroimaging. To overcome this limitation, a data-driven analysis called functional connectivity density (FCD) mapping was proposed, treating each voxel as a seed and calculating the number of connections it holds with other voxels, thereby indirectly elucidating the spatial distribution and importance of brain regions within the whole brain [7]. Thus, the FCD provides an unbiased search of functional connectome abnormalities within the whole brain without prior hypothesis [8]. This method has been used to detect functional reorganization patterns across diverse diseases, such as stroke [2, 9, 10], depression [11], and Parkinson’s disease [12].

Increasing evidence has indicated that the FC between brain regions is closely associated with their anatomical locations [13, 14]. Recent studies divided the FCD into short-range FCD (sFCD) and long-range FCD (lFCD) based on a distance criterion [15], representing functional specialization and integration of brain networks, respectively [16]. This approach has identified distance-dependent FCD alterations in neuropsychiatric disorders. For instance, a schizophrenia study revealed a significant interaction between genotype and diagnosis in the sFCD but not in the lFCD [17]. Another study found altered lFCD, but not altered sFCD, in the frontoparietal areas in children with attention-deficit/hyperactivity disorder [18]. The bipolar disorder patients showed a positive correlation between increased sFCD in the left fusiform gyrus and depressive episodes and a negative correlation between decreased lFCD in the left angular gyrus and depressive severity [19]. The major depressive patients displayed significantly negative correlations between decreased sFCD in the left precentral /postcentral gyrus and depressive severity, which were not observed in the lFCD [20]. However, previous FCD studies in stroke have not accounted for the effect of distance on functional reorganization, and it remains unclear whether the FCD shows distance-dependent alterations in non-acute stroke patients.

The present study aimed to explore whether the functional reorganization in cerebral and cerebellar cortices correlates with the spatial distances between brain regions in non-acute stroke patients following left and right hemisphere subcortical lesions using the FCD approach. Based on the prior reports on stroke [4–6, 21], we hypothesized that non-acute stroke patients with unilateral subcortical lesions would show distance-dependent FCD changes in motor and non-motor brain regions, such as sensorimotor cortex, frontoparietal cortex, and cerebellum, which would correlate with the motor function of the patients and might serve as potential biomarkers to predict motor outcome after stroke.

## Methods

### Participants

The present study was conducted between January 2022 and September 2023. A cohort of 64 patients who had suffered from a unilateral non-acute stroke and 41 HCs were initially recruited from the Nanjing Drum Tower Hospital, Affiliated Hospital of Medical School, Nanjing University, and residential areas adjacent to the hospital. The study was structured into two components: a cross-sectional investigation involving 44 patients and all HCs, and a longitudinal experiment with 10 patients. The inclusion criteria for stroke patients were as follows: (1) confirmation of first-episode, unilateral, subcortical stroke using CT or MRI; (2) age > 18 years; (3) right-handedness prior to the stroke event; (4) stroke duration > 3 months since the onset of stroke; and (5) normal or corrected-to-normal hearing and vision. Patients were excluded if they met any of the following conditions: (1) contraindication for MRI; (2) coexistence of neuropsychiatric disorders other than stroke, including but not limited to anxiety disorders, major depressive disorders, schizophrenia, and bipolar disorder; (3) unstable medical conditions, such as severe atrial fibrillation; (4) prior exposure to transcranial electromagnetic/ultrasound stimulation [22]; and (5) a history of tobacco, alcohol, or other drug addiction since the onset of stroke. The unilateral stroke patient cohort in cross-sectional dataset was further subdivided into two subgroups based on the location of the subcortical lesion: left subcortical stroke (LSS) and right subcortical stroke (RSS) groups. As for the inclusion criteria for the HCs group, participants were required to satisfy the following conditions: (1) closely matched age and educational levels with the stroke patient cohort, and (2) right-handedness. Exclusion criteria for the HCs group entailed the presence of (1) noticeable physical or neuropsychiatric disorders and (2) a history of tobacco, alcohol, or other drug addiction.

The sample size in the current study was determined according to the reports in the previous studies investigating functional reorganization after stroke [23, 24]. Following the exclusion of incomplete MRI scans (3 LSS patients, 2 RSS patients, and 2 HCs) and the presence of excessive head motion (2 LSS patients, as elaborated in the Data preprocessing), a final cohort of 25 LSS patients, 22 RSS patients, and 39 HCs constituted the cross-sectional dataset for subsequent analysis. A power analysis was calculated for a one-way analysis of covariance (ANCOVA) using the G*Power tool [25]. With an effect size of 0.5 (which indicates a large statistical power [25]), *α* set at 0.05, a total sample size of 86 participants, and three groups under consideration, the power (1 − *β*) stood at 0.95.

### Behavioral instruments

Before MRI scanning, each stroke patient underwent evaluations of motor performance and activities of daily living utilizing the Fugl-Meyer Assessment (FMA) and the Chinese version of Modified Barthel Index (MBI-C), respectively. For non-acute stroke patients, achieving a score of 9 (sensitivity: 80.39%, specificity: 70%) up to 10 (sensitivity: 97.62%, specificity: 89.66%) on the FMA Upper Extremity (FMA-UE) scale indicates a higher likelihood of experiencing clinical improvement in disability [26]. Furthermore, the FMA Lower Extremity (FMA-LE) scale demonstrates commendable sensitivity (0.87) and specificity (0.81) in differentiating levels of lower extremity function among chronic stroke survivors [27]. The MBI-C measures the activities of daily living of stroke survivors and can be categorized into functional performance and physiological needs. Notably, the MBI-C exhibits comparable validity and reliability to the original version at the item level, with kappa statistics ranging from 0.63 to 1.00 [28].

It is noteworthy that 7 patients in the longitudinal dataset underwent two identical behavioral assessments with an average interval of 20 days, during which the patients received routine rehabilitation treatments, such as physical and/or occupational therapy.

### MRI data acquisition

All MRI data were acquired at a 3.0 T MRI scanner (Philips Healthcare, Netherlands). Resting-state fMRI was scanned using an echo-planar imaging sequence with the following parameters: repetition time = 2000 ms, echo time = 30 ms, matrix = 64 × 64, slice thickness = 4 mm, field of view = 192 mm × 192 mm, voxel size = 3 mm × 3 mm × 4 mm, flip angle = 90°, 38 axial slices, 230 volumes, and scan time = 8 min 08 s. Furthermore, three-dimensional high-resolution T1-weighted images were obtained using a three-dimensional fast field-echo sequence with the following parameters: repetition time = 9.9 ms, echo time = 4.6 ms, matrix = 256 × 256, slice thickness = 1 mm, field of view = 256 mm × 256 mm, 192 sagittal slices, voxel size = 1 mm × 1 mm × 1 mm, flip angle = 8°, and scan time = 6 min 47 s. Additionally, T2-weighted images were collected using a MultiVane sequence with the following parameters: repetition time = 4000 ms, echo time = 91 ms, matrix = 230 × 230, slice thickness = 5 mm, field of view = 230 mm × 230 mm, 30 axial slices, voxel size = 1 mm × 1 mm × 5 mm, flip angle = 90°, and scan time = 1 min 4 s. Notably, 7 patients in the longitudinal dataset underwent two MRI scans with an averaged interval of 20 days.

### Lesion overlap analysis

**Fig. 1:**
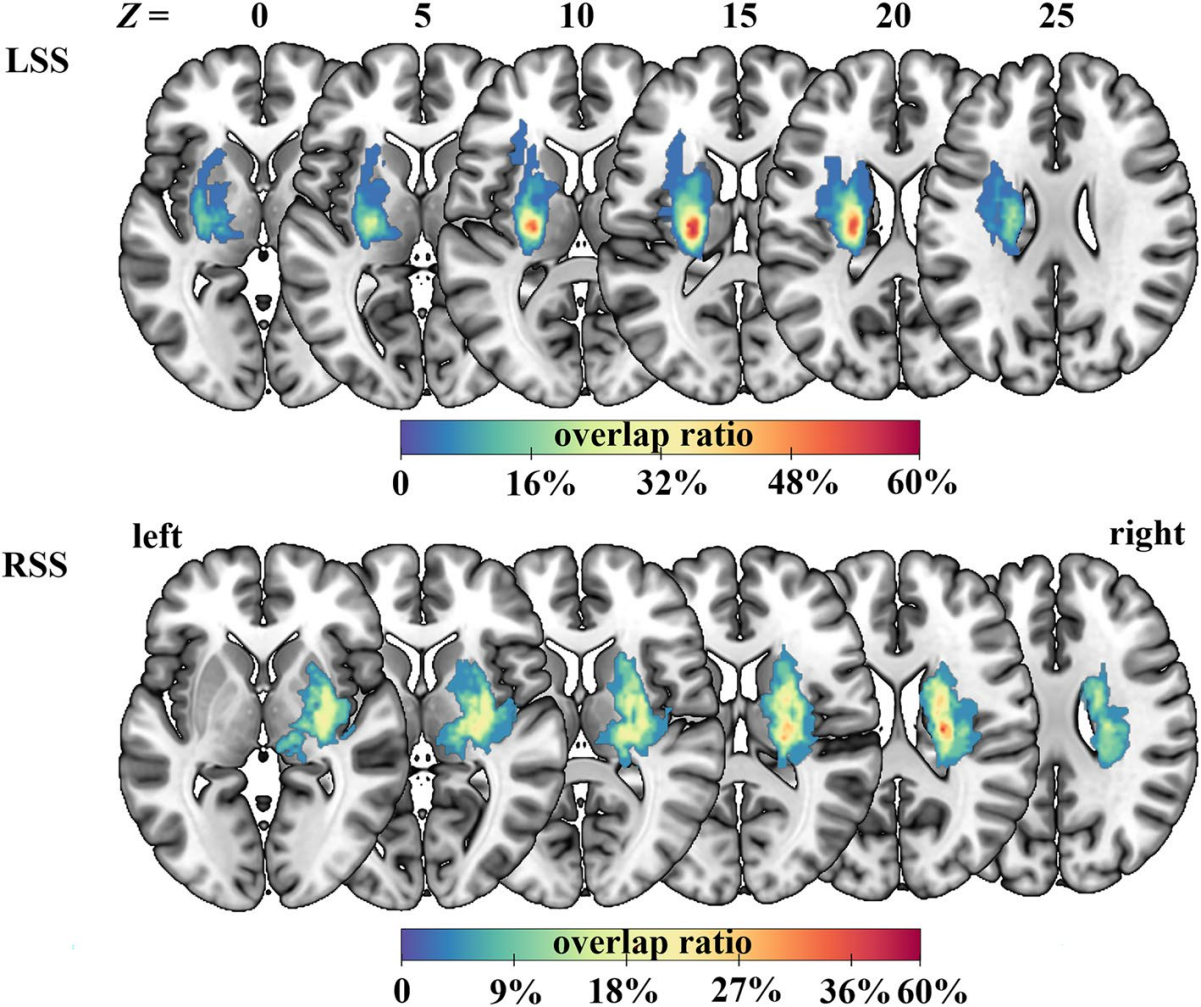
Lesion overlap map for LSS and RSS patients. The color bar indicates the frequency of patients with lesions in each voxel. LSS, left subcortical stroke; RSS, right subcortical stroke

Using MRIcron software (https://www.nitrc.org/projects/mricron), two physicians, who were blinded to the clinical data, determined the lesion outline on T2-weighted images for each stroke patient. Then, the lesion masks of all stroke patients were normalized to the Montreal Neurological Institute space based on the echo-planar imaging template. Finally, all the normalized lesion masks were summed to generate a lesion overlap map within each patient subgroup. The group-level lesion overlap map and the precise lesion locations for each patient are displayed in Fig. 1 and Additional file 1: Fig. S1, respectively.

### Data preprocessing

Resting-state fMRI data were preprocessed using the *Advanced* Data Processing Assistant for Resting-State fMRI (DPARSF) software (http://rfmri.org/DPARSF) [29]. The preprocessing procedure included the following steps: (1) removal of the first 10 volumes; (2) slice-timing correction; (3) head motion correction, with the exclusion of 2 LSS patients who exhibited excessive motion exceeding 2.5 mm of translation or greater than 2.5 degrees of rotation in any direction; (4) regression of the linear trend, white matter and cerebrospinal fluid signals, and the 24 head motion parameters [30]; (5) spatial normalization using a diffeomorphic anatomical registration through exponentiated lie algebra (DARTEL) method, and resampled every 3 mm; (6) spatial smoothing with a full width at a half maximum (FWHM) = 6 mm, and (7) temporal bandpass filtering (0.01–0.1 Hz).

### Distance-dependent FCD analysis

To explore the effect of distance on functional connections, the three-dimensional anatomical distance between every pair of voxels (*i* and *j*) was approximated using the Euclidean distance:

$${D}_{ij}= \sqrt{{({x}_{i}-{x}_{j})}^{2}+{({y}_{i}-{y}_{j})}^{2}+{({z}_{i}-{z}_{j})}^{2}}$$

where (x_*i*_, y_*i*_, z_*i*_) and (x_*j*_, y_*j*_, z_*j*_) are stereotaxic coordinates for voxels *i* and *j*, respectively, in the Montreal Neurological Institute space. The FCD of a voxel indicates the average strength of its functional connection with all other voxels. The functional connections of each voxel were classified as either short- or long-range based on a distance criterion of 12 mm [13, 31]. To improve normality, the FCD maps were converted to *z*-values using Fisher’s *r*-to-*z* transformation. Here, the FCD was calculated using the absolute weighted value of the functional connection. Finally, each subject yielded three *z*-maps representing global, long-range, and short-range FCD (gFCD/lFCD/sFCD).

### Statistical analyses

Statistical analyses were conducted using the Statistical Package for Social Sciences (SPSS) version 21 for Windows to compare the demographic characteristics and clinical assessments between LSS, RSS, and HCs in the cross-sectional dataset, and between pre- and post-observations in the longitudinal dataset.

In the cross-sectional dataset, we first performed ANCOVA analysis to compare differences in FCD values among the three groups, controlling for age, gender, education, and head motion as covariates (with Gaussian Random Field (GRF) correction, voxel-level *P* < 0.01, and cluster-level *P* < 0.05, two-tailed). Then, post hoc two-sample *t*-tests were conducted to explore differences between all paired groups. Moreover, a linear regression analysis was employed to assess the relationship between the FCD values and the scores on clinical assessments (FMA-UE, FMA-LE, and MBI-C scales) while controlling for age, gender, education, head motion, and lesion volume in the cross-sectional dataset. This regression analysis was conducted separately for the LSS, RSS, and total patients, with false discovery rate (FDR) correction (*P* < 0.05).

### Validation analysis

Considering the relatively small sample size of each group in the current study, we performed three distinct validations as follows: (1) we recalculated the lFCD and sFCD using alternative distance thresholds of 6 mm and 18 mm, respectively; (2) we carried out a leave-one-out cross-validation methodology [32]. Specifically, one stroke patient was left out of the sample, and the ANCOVA analysis was performed on permuted datasets (i.e., 24 LSS vs. 22 RSS vs. 39 HCs or 25 LSS vs. 21 RSS vs. 39 HCs). The leave-one-out cross-validation resulted in a total of 47 *F* maps, which were then used to compute voxel-wise reproducibility by calculating the number of a voxel that exhibited significant differences among the three groups across 47 ANCOVA tests; and (3) we validated the associations between FCD values and clinical assessments in the longitudinal dataset.

## Results

### Demographic characteristics and clinical assessment

For the cross-sectional dataset, 9 participants were excluded due to incomplete MRI scans for personal reasons (3 LSS patients, 2 RSS patients, and 2 HCs) and excessive head motion (2 LSS patients). The final analysis included 25 LSS patients, 22 RSS patients, and 39 HCs. These groups were well-matched in age, gender, education level, and head motion (*P* > 0.05). For the longitudinal dataset, 3 patients were excluded from the analysis due to incomplete the second session of MRI scans for personal reasons. The detailed demographic characteristics and clinical assessments for both stroke patients and HCs are presented in Table 1.

**Table 1:** Demographics and clinical details of the participants

	Cross-sectional dataset					Longitudinal dataset	
	LSS group (*n* = 25)	RSS group (*n* = 22)	HCs group (*n* = 39)	*F/t*/*χ*^2^ value	*P* value	(*n* = 7)	
Age (years)	57.72 ± 10.68	54.55 ± 9.15	57.90 ± 8.50	1.01	0.37^a^	52.43 ± 6.53	
Gender (male:female, *n*)	22:3	19:3	35:4	0.16	0.92^b^	6: 1	
Education (years)	10.40 ± 2.77	9.86 ± 2.75	9.92 ± 3.40	0.24	0.79^a^	11.29 ± 3.09	
Duration of illness (months)	13.47 ± 10.32	14.02 ± 10.62	-	− 0.18	0.86^a^	14.86 ± 12.62	
Lesion volume (ml)	2.58 ± 1.92	3.39 ± 3.59	-	− 0.97	0.34^a^	3.92 ± 4.68 (pre)	3.70 ± 4.86 (post)
Head motion (mm)	0.17 ± 0.18	0.14 ± 0.08	0.13 ± 0.11	0.82	0.44^a^	0.11 ± 0.04 (pre)	0.12 ± 0.05 (post)
FMA-UE	44.40 ± 19.18	46.59 ± 17.44	-	− 0.41	0.69^a^	31.43 ± 9.54 (pre)	36.14 ± 10.89 (post)
FMA-LE	27.96 ± 5.41	27.68 ± 6.82	-	0.16	0.88^a^	29.29 ± 3.73 (pre)	31.00 ± 2.94 (post)
MBI-C	79.20 ± 22.17	87.73 ± 14.41	-	− 1.58	0.12^a^	72.86 ± 5.31 (pre)	76.57 ± 5.32 (post)

*LSS* Left subcortical stroke, *RSS* Right subcortical stroke, *HCs* Healthy controls, *FMA-UE* Fugl-Meyer Assessment of Upper Extremity, *FMA-LE* Fugl-Meyer Assessment of Lower Extremity, *MBI-C* the Chinese version of Modified Barthel Index

^a^ANOVA for three groups and Independent *t*-test for two groups in a cross-sectional study

^b^Chi-square test in a cross-sectional study

#### Comparisons between groups in the FCD

**Fig. 2:**
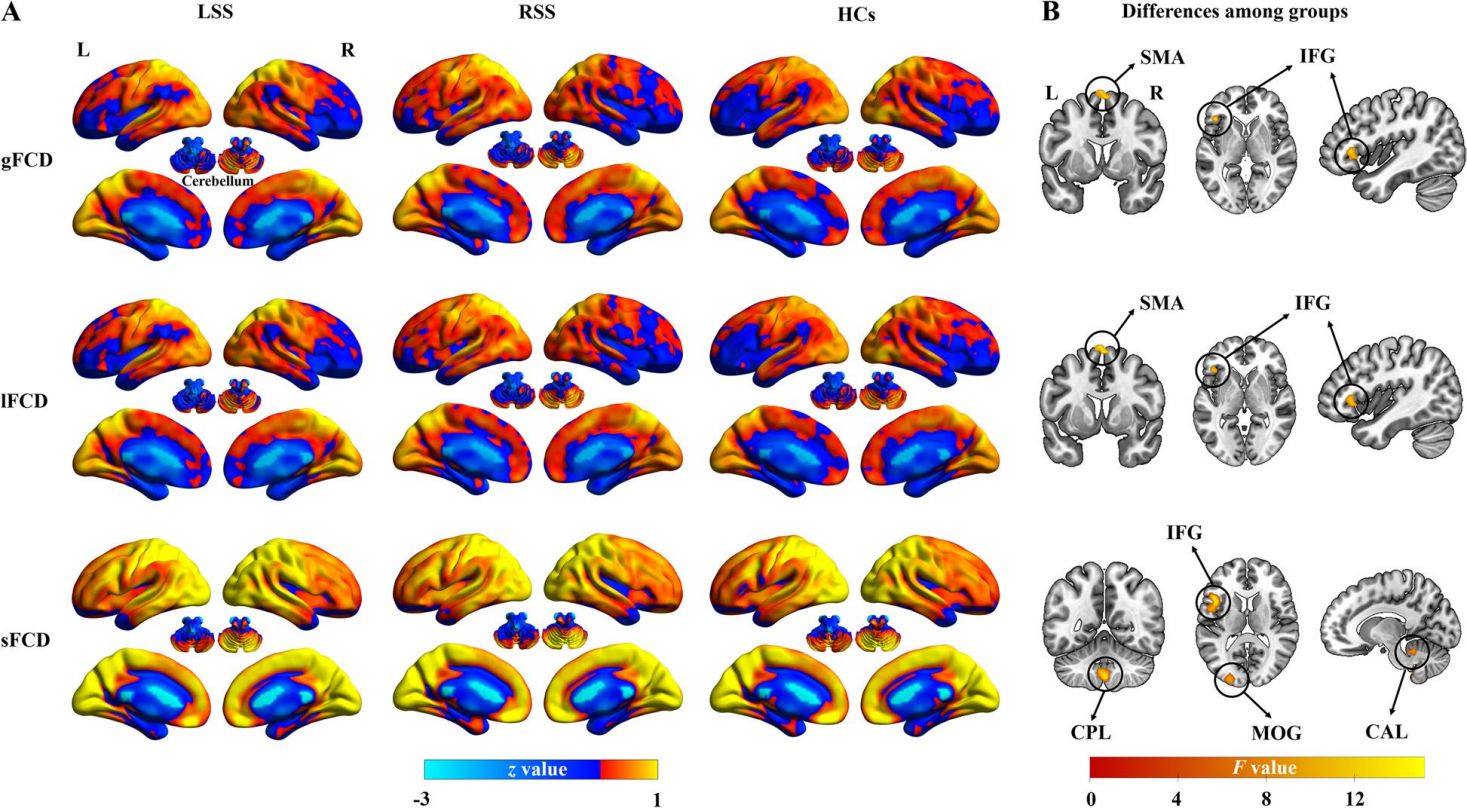
FCD maps in each group and their differences among the three groups. A represents the mean FCD map in each group and B represents significant differences in FCD among LSS, RSS, and HCs. LSS, left subcortical stroke; RSS, right subcortical stroke; gFCD, global functional connectivity density; lFCD, long-range functional connectivity density; sFCD, short-range functional connectivity density; SMA, supplementary motor area; IFG, inferior frontal gyrus; CPL, cerebellum posterior lobe; MOG, middle occipital gyrus; CAL, cerebellum anterior lobe

**Fig. 3:**
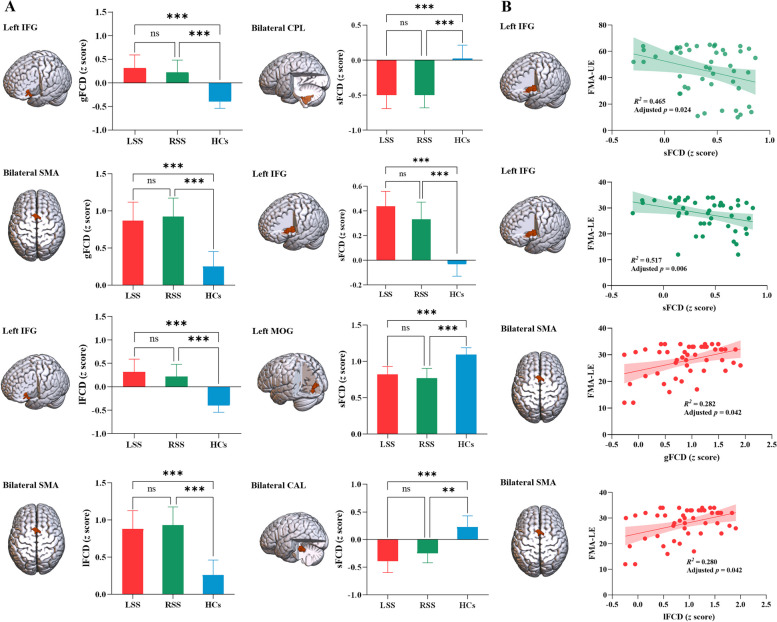
Significant differences between LSS/RSS and HCs in FCD and their correlations with motor performance. A represents regions showing significant differences among LSS, RSS, and HCs and B represents correlations between altered FCD patterns and hemiplegic motor performance across all stroke patients. LSS, left subcortical stroke; RSS, right subcortical stroke; gFCD, global functional connectivity density; lFCD, long-range functional connectivity density; sFCD, short-range functional connectivity density; SMA, supplementary motor area; IFG, inferior frontal gyrus; CPL, cerebellum posterior lobe; MOG, middle occipital gyrus; CAL, cerebellum anterior lobe; FMA-UE, Fugl-Meyer Assessment Upper Extremity Scale; FMA-LE, Fugl-Meyer Assessment Lower Extremity Scale. *, 0.01 < *P* < 0.05; **, 0.001 < *P* < 0.01; ***, *P* < 0.001; ns, not significant

Figure 2A displays the mean FCD maps for each group. At the whole-brain level, the LSS group exhibited significantly higher mean values of gFCD (*P* = 0.001, Cohen’s *d* = 0.89), lFCD (*P* = 0.001, Cohen’s *d* = 0.89), and sFCD (*P* = 0.002, Cohen’s *d* = 0.83) compared to the HCs (Additional file 1: Fig. S2). However, no significant differences in the three FCD values were found between other paired groups (*P* > 0.05). At the voxel level, the ANCOVA analysis showed significant group differences in the left inferior frontal gyrus and bilateral supplementary motor gyrus for both gFCD and lFCD (Table 2 and Fig. 2B), and in the left inferior frontal gyrus, bilateral cerebellum posterior lobe, left middle occipital gyrus, and bilateral cerebellum anterior lobe for sFCD (Table 2 and Fig. 2B). Post hoc analyses subsequently revealed that both the LSS and RSS groups exhibited significantly increased gFCD and lFCD values in the left inferior frontal gyrus and bilateral supplementary motor gyrus when compared to the HCs (Fig. 3A, |Cohen’s *d|*> 0.8 in Table 2). Moreover, both the LSS and RSS groups demonstrated increased sFCD values in the left inferior frontal gyrus and precentral gyrus, and decreased sFCD values in the bilateral cerebellum posterior lobe, left middle occipital gyrus, and bilateral cerebellum anterior lobe, in comparison to the HCs (Fig. 3A, |Cohen’s *d|*> 0.8 in Table 2). However, no significant differences in gFCD, lFCD, or sFCD values were observed when comparing the two patient subgroups (Table 2). Collectively, the analyses revealed that the left inferior frontal gyrus showed distance-independent FCD alteration, while bilateral supplementary motor gyrus, cerebellum anterior lobe, cerebellum posterior lobe, and left middle occipital gyrus exhibited distance-dependent FCD alterations in stroke patients.

**Table 2:** Regions showing significant differences in gFCD, lFCD, and sFCD among LSS, RSS, and HCs

Regions	Hemisphere	MNI coordinates			Cluster size	*F* value	LSS vs. HCs		RSS vs. HCs		LSS vs. RSS	
		*x*	*y*	*z*			*t* value	Cohen’s *d*	*t* value	Cohen’s *d*	*t* value	Cohen’s *d*
*gFCD*												
Inferior frontal gyrus	Left	− 45	27	3	39	11.29	4.61***	1.23	4.54***	1.17	0.50	0.15
Supplementary motor area	Bilateral	9	3	72	23	9.29	3.96***	1.02	4.24***	1.15	− 0.32	− 0.09
*lFCD*												
Inferior frontal gyrus	Left	− 45	27	3	39	11.31	4.62***	1.23	4.53***	1.17	0.51	0.15
Supplementary motor area	Bilateral	9	3	72	22	9.27	3.98***	1.02	4.25***	1.15	− 0.31	− 0.09
*sFCD*												
Cerebellum posterior lobe	Bilateral	0	− 54	− 51	73	14.33	− 3.77***	− 0.99	− 3.74***	− 1.05	0.03	0.01
Inferior frontal gyrus	Left	− 45	18	12	57	16.24	6.11***	1.57	4.43***	1.17	1.19	0.35
Precentral gyrus	Left				27							
Middle occipital gyrus	Left	− 24	− 93	12	69	10.47	− 3.84***	− 0.99	− 4.14***	− 1.10	0.63	0.19
Cerebellum anterior lobe	Bilateral	3	− 33	− 21	37	12.39	− 4.19***	− 1.10	− 3.67**	− 0.91	− 1.11	− 0.33

*MNI* Montreal Neurological Institute, *LSS* Left subcortical stroke, *RSS* Right subcortical stroke, *HCs* Healthy controls, *gFCD* global functional connectivity density, *lFCD* Long-range functional connectivity density, *sFCD* short-range functional connectivity density

*, 0.01 < *P* < 0.05; **, 0.001 < *P* < 0.01; ***, *P* < 0.001

### Correlations between FCD and clinical assessments

**Fig. 4:**
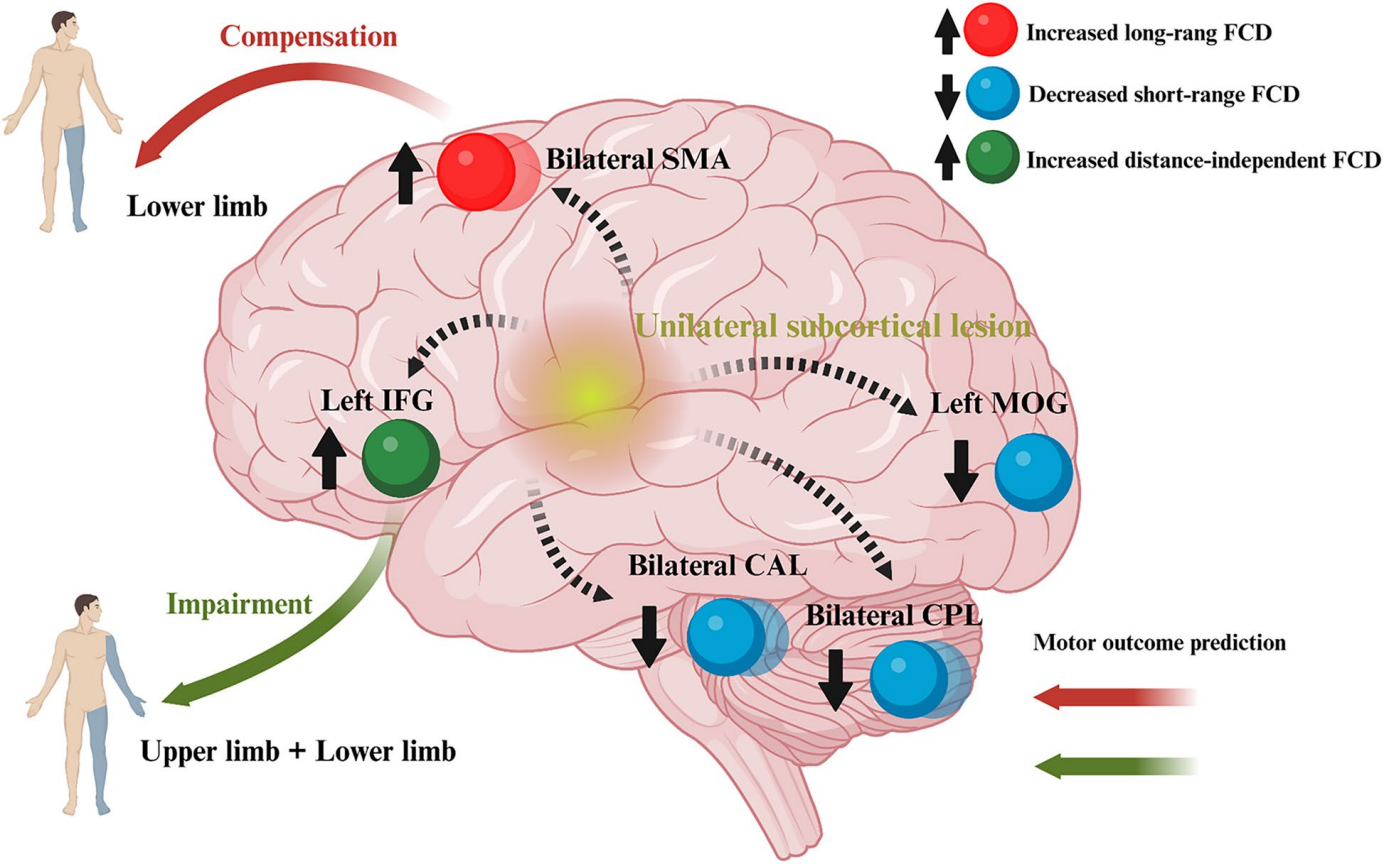
Distance-related cortical functional reorganization after subcortical stroke. FCD, functional connectivity density; SMA, supplementary motor area; IFG, inferior frontal gyrus; CPL, cerebellum posterior lobe; MOG, middle occipital gyrus; CAL, cerebellum anterior lobe. Created with BioRender.com

**Fig. 5:**
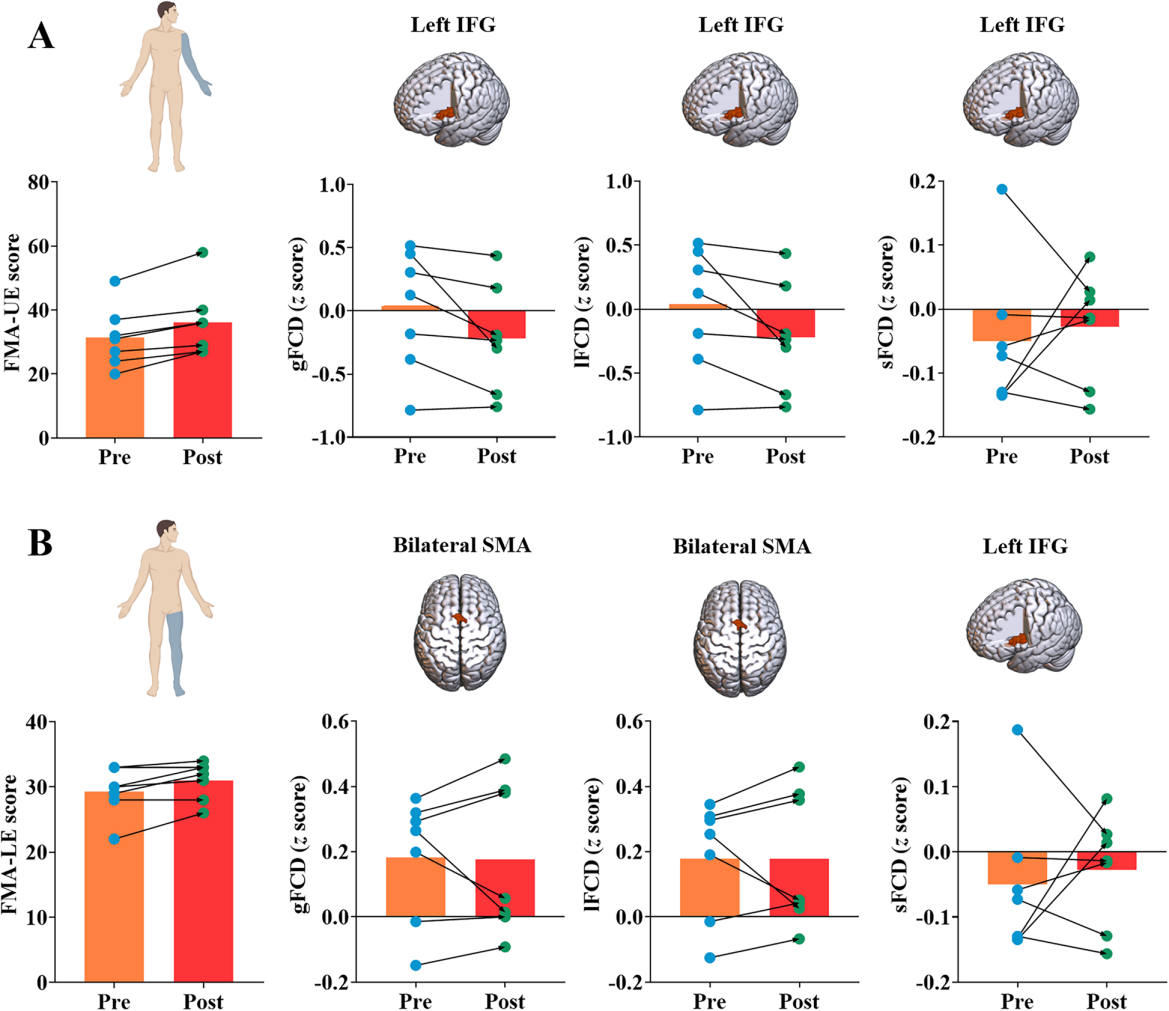
The changes of FCD and motor outcome in stroke patients after routine intervention. A represents the FCD alterations related to FMA-UE and B represents the FCD alterations related to FMA-LE. IFG, inferior frontal gyrus; SMA, supplementary motor area; gFCD, global functional connectivity density; lFCD, long-range functional connectivity density; sFCD, short-range functional connectivity density; FMA-UE, Fugl-Meyer Assessment Upper Extremity Scale; FMA-LE, Fugl-Meyer Assessment Lower Extremity Scale

The sFCD in the left inferior frontal gyrus showed negative correlations with FMA-UE (*β* =  − 0.007, adjusted *P* = 0.024) and FMA-LE (*β* =  − 0.025, adjusted *P* = 0.006) scores, and both gFCD (*β* =  − 0.011, adjusted* P* = 0.130) and lFCD (*β* =  − 0.011, adjusted* P* = 0.130) in this region negatively correlated with FMA-UE score across all stroke patients (Table 3 and Fig. 3B). Furthermore, positive correlations were observed between gFCD (*β* = 0.040, adjusted *P* = 0.042) and lFCD (*β* = 0.040, adjusted *P* = 0.042) in the bilateral supplementary motor gyrus and the FMA-LE scores across all stroke patients (Table 3 and Fig. 3B). There were no significant correlations between the FCD and the motor outcomes in LSS or RSS group (adjusted *P* > 0.05, Table 3). Detailed information is presented in Fig. 4. More importantly, the significant correlations aforementioned were validated by a longitudinal dataset. After routine intervention, the scores of upper/lower extremities increased in all patients, while FMA-UE-related FCD in the inferior frontal gyrus decreased in > 4/7 of patients (Fig. 5A) and FMA-LE-related FCD in the supplementary motor gyrus decreased in > 5/7 of patients (Fig. 5B).

**Table 3:** Correlations between FCD values and motor-related outcomes across all post-stroke patients

Regions	Feature	*β*	*R*^2^	*t*	*P*	Adjusted *P*
*FMA-UE*						
Left IFG	gFCD	− 0.011	0.202	− 2.152	0.038	0.130
Bilateral SMA	gFCD	0.008	0.202	1.804	0.079	0.157
Left IFG	lFCD	− 0.011	0.202	− 2.154	0.037	0.130
Bilateral SMA	lFCD	0.008	0.199	1.826	0.075	0.157
Bilateral CPL	sFCD	0.005	0.279	1.435	0.159	0.254
Left IFG	sFCD	− 0.007	0.465	− 3.301	0.002	0.024
Left MOG	sFCD	0.235	0.189	1.028	0.310	0.465
Bilateral CAL	sFCD	− 0.900	0.134	1.717	0.094	0.161
*FMA-LE*						
Left IFG	gFCD	− 0.031	0.181	− 1.868	0.069	0.157
Bilateral SMA	gFCD	0.040	0.282	2.844	0.007	0.042
Left IFG	lFCD	− 0.031	0.180	− 1.867	0.069	0.157
Bilateral SMA	lFCD	0.040	0.280	2.286	0.007	0.042
Bilateral CPL	sFCD	0.010	0.257	0.906	0.370	0.522
Left IFG	sFCD	− 0.025	0.517	− 4.035	< 0.001	0.006
Left MOG	sFCD	0.002	0.169	0.317	0.753	0.786
Bilateral CAL	sFCD	0.009	0.083	0.740	0.464	0.554
*MBI-C*						
Left IFG	gFCD	− 0.009	0.174	− 1.764	0.085	0.157
Bilateral SMA	gFCD	0.003	0.148	0.704	0.485	0.554
Left IFG	lFCD	− 0.009	0.174	− 1.768	0.085	0.1569
Bilateral SMA	lFCD	0.003	0.144	0.712	0.481	0.554
Bilateral CPL	sFCD	0.001	0.245	0.395	0.695	0.758
Left IFG	sFCD	− 0.005	0.413	− 2.516	0.016	0.077
Left MOG	sFCD	< 0.001	0.167	0.101	0.920	0.920
Bilateral CAL	sFCD	0.003	0.087	0.862	0.394	0.525

*FMA-UE* Fugl-Meyer Assessment Upper Extremity Scale, *FMA-LE* Fugl-Meyer Assessment Lower Extremity Scal, *MBI-C* the Chinese version of Modified Barthel Index, *IFG* Inferior frontal gyrus, *SMA* Supplementary motor area, *CPL* Cerebellum posterior lobe, *MOG* Middle occipital gyrus, *CAL* Cerebellum anterior lobe, *gFCD* global functional connectivity density, *lFCD* Long-range functional connectivity density, *sFCD* short-range functional connectivity density

### Validation results

Between-group differences in gFCD, lFCD, and sFCD among LSS, RSS, and HCs under two different distance criteria (6 mm and 18 mm) replicated the primary findings observed under the 12 mm criterion (Additional file 1: Table S1 and Table S2). Namely, the left inferior frontal gyrus continued to exhibit distance-dependent FCD alteration, and the bilateral supplementary motor gyrus and cerebellum posterior lobe consistently displayed distance-independent FCD alteration in both patient subgroups compared with HCs. Also, the post hoc analyses between the two patient subgroups and HCs showed large effect sizes, with |Cohen’s *d|*> 0.8 (Additional file 1: Table S1 and Table S2). Moreover, the leave-one-out cross-validation indicated the high reproducibility of FCD differences observed among the three groups, as shown in Additional file 1: Fig. S3.

## Discussion

The present study used the distance-dependent FCD approach to investigate functional alterations in non-acute stroke patients following left and right hemisphere subcortical lesions. Our results demonstrated that, compared with HCs, the LSS and RSS groups both exhibited distance-dependent FCD changes, that bilateral supplementary motor gyrus showed increased gFCD and lFCD, while bilateral cerebellum anterior/posterior lobe and left middle occipital gyrus showed reduced sFCD. Interestingly, the left inferior frontal gyrus showed a distance-independent FCD increase in both patient subgroups compared to HCs. Moreover, we found significant correlations between the FCD values in the left inferior frontal gyrus as well as bilateral supplementary motor gyrus and specific motor functions in stroke patients. These findings support our initial hypothesis, suggesting the presence of distance-related functional reorganization in motor and non-motor regions of non-acute subcortical stroke, which may serve as valuable biomarkers for predicting motor outcomes in stroke patients.

### Abnormal global FCD in stroke groups

Numerous studies have utilized gFCD to explore neural activity alterations after stroke. For instance, Min et al. found higher gFCD values in the right parahippocampal gyrus in acute subcortical stroke patients than HCs [9]. Wang et al. demonstrated that subacute stroke patients with cognitive impairment exhibited significant changes in gFCD, including decreases in language-related brain regions and increases in the right middle frontal gyrus, hippocampus, and paracentral lobule, in comparison to HCs [2]. Yao et al. reported decreased gFCD values in the right inferior parietal lobule and right postcentral gyrus, in subacute stroke patients compared with HCs [10]. Different from the acute and subacute stroke, the present study found that two non-acute stroke subgroups both showed increased gFCD in the bilateral supplementary motor gyrus compared with HCs. As a dominant region contributing to movement control, the supplementary motor gyrus participates in stabilizing body posture and regulating movement sequences [33]. Previous studies found that the supplementary motor gyrus showed higher functional connectivity [34] and increased regional homogeneity [35] in subcortical stroke patients than HCs, indicating a compensatory role of the supplementary motor gyrus in response to motor deficits after stroke. Therefore, our findings align with previous reports and further demonstrated that the supplementary motor gyrus plays an important role underlying the functional reorganization in stroke patients.

### Disrupted distance-related FCD in the stroke groups

Previous studies have demonstrated that schizophrenia leads to abnormal sFCD rather than the lFCD in the precuneus [17], and attention-deficit/hyperactivity disorder alters the lFCD but not the sFCD in the left superior parietal gyrus and the right middle frontal gyrus [18]. Patients with cognitive impairment showed increased lFCD in the left fusiform gyrus and sFCD in the left middle orbital gyrus [36]. Similarly, such distance-dependent patterns were observed in the present study that the bilateral supplementary motor gyrus and left inferior frontal gyrus showed increased lFCD, and the bilateral cerebellum anterior/posterior lobe and left middle occipital gyrus showed decreased sFCD in non-acute subcortical stroke patients following left and right hemisphere subcortical lesions compared to HCs. The functional reorganizations in these regions have been reported in stroke patients, who showed decreased regional homogeneity [37] and interhemispheric connectivity [10] in the middle occipital gyrus and supplementary motor gyrus, as well as increased FC in the cerebellum anterior lobe [34] and the cerebellum posterior lobe [38] compared to HCs. A recent study found an increased FCD value of the right cerebellum posterior lobe in stroke patients relative to HCs [2]. Therefore, the current study further extended the existing knowledge by emphasizing that function reorganization in the frontal-occipital cortex and cerebellum exhibits a distance-dependent pattern following subcortical stroke regardless of the lesion hemisphere. Indeed, lFCD and sFCD reflect the functional integration and specialization of brain networks, respectively [15, 16, 18]. Our findings suggest that non-acute subcortical stroke patients exhibit long-range functional plasticity (compensatory effect) in the supplementary motor gyrus, while the regional functional plasticity is primarily present in the cerebellum and middle occipital gyrus, highlighting the intricacy and flexibility of functional reorganization during the non-acute phase post subcortical stroke.

### Distance-independent FCD increase in the left inferior frontal gyrus after stroke

In addition to its traditional roles in cognitive processes [39] and speech functions [40], the inferior frontal gyrus assumes a core component in the mirror neuron system [41], which is closely associated with action observation [42] and motion imitation [43]. Furthermore, the inferior frontal gyrus has been implicated in specific motor-related processes, including motor imagery [44] and task execution [5]. Multiple studies have focused on the functional plasticity of the inferior frontal gyrus in stroke patients with motor dysfunction. For instance, Garrison et al. reported cortical activations in the bilateral inferior frontal gyrus during hand movement in chronic stroke patients [45]. Ma et al. found increased activation in the inferior frontal gyrus during motor imagery in subacute subcortical stroke patients compared to HCs [46]. Furthermore, diverse functional alterations in the inferior frontal gyrus have been documented in stroke patients. Compared with HCs, stroke patients showed increased FCD in the bilateral inferior frontal-orbital gyrus [37] and decreased interhemispheric connectivity in the bilateral inferior frontal gyrus [5, 10]. Consistently, we found increased gFCD, lFCD, and sFCD in the left inferior frontal gyrus in non-acute subcortical stroke patients with motor impairment, compared to HCs, indicating a distance-independent functional reorganization in this region. Interestingly, our findings remain consistent regardless of the lesion hemisphere, and it should be noted that the patients recruited in our study did not exhibit aphasia. To provide a more comprehensive understanding of the post-stroke functional changes in the left inferior frontal gyrus, the present study selected the left inferior frontal gyrus as the seed region for a whole-brain FC analysis and found FC alterations between the left inferior frontal gyrus and motor-related regions, such as the right precentral gyrus, postcentral gyrus, middle frontal gyrus, bilateral middle temporal gyrus, and left precuneus (Additional file 1: Fig. S4 and Table S3), which have previously been implicated in stroke patients [5, 47]. Thus, we revealed a reorganization of functional networks with the left inferior frontal gyrus as the hub following subcortical stroke, thereby providing evidence for the critical role of the left inferior frontal gyrus in motor function.

In summary, both the LSS and RSS consistently showed distance-related FCD changes. Specifically, the left inferior frontal gyrus displayed distance-independent FCD alterations, whereas the bilateral supplementary motor gyrus, cerebellum anterior/posterior lobe, and left middle occipital gyrus exhibited distance-dependent FCD alterations. Considering the correlations between FCD and motor outcome, the increased FCD in the bilateral supplementary motor gyrus may play a compensatory role in the restoration of motor function in the paralyzed lower limbs of stroke patients, while the increased FCD in the left inferior frontal gyrus may indicate impairment of motor function in both the paralyzed upper and lower limbs of stroke patients.

### Association of functional reorganization with motor outcomes

Previous studies have reported a positive correlation between voxel-mirrored homotopic connectivity in the inferior frontal gyrus and motor function in Parkinson’s disease [48], and a negative correlation between the lesion size of the left inferior frontal gyrus and hand action performance in chronic stroke patients [49]. Moreover, subacute stroke patients showed significant positive correlations between the variability of amplitude of low-frequency fluctuation in the supplementary motor gyrus and total FMA scores [50], and between functional connectivity in the supplementary motor gyrus and FMA-UE scores [51]. Chronic stroke patients exhibited a significantly positive correlation between neural complexity values in the supplementary motor gyrus and total FMA scores [52]. These findings underscore the close association between functional alterations in the inferior frontal gyrus and supplementary motor gyrus and motor dysfunction following stroke. The present study utilized a cross-sectional dataset comprising subjects with varying durations of illness, resembling a pseudo-longitudinal dataset. This approach found that the sFCD value in the left inferior frontal gyrus significantly negatively correlated with the motor function in both upper and lower extremities, while the gFCD and lFCD values in the bilateral supplementary motor gyrus significantly positively correlated with the FMA-LE scores, suggesting the predictive potential of FCD for motor outcomes. Additionally, we gathered a longitudinal dataset with two time points to confirm these relationships, revealing a predictive relationship reliability of over 70%, consistent with the experimental design reported in the previous studies [53, 54]. Our findings indicate that the increased FCD in the bilateral supplementary motor gyrus may function as a compensatory mechanism [55, 56] in the recovery of motor function in the paralyzed lower limbs of stroke patients, whereas increased FCD in the left inferior frontal gyrus may indicate impairment [49] in motor function in both the paralyzed upper and lower limbs of stroke patients. Taken together, this suggests that the FCD values in the left inferior frontal gyrus and bilateral supplementary motor gyrus could serve as biomarkers for predicting motor outcomes after stroke.

### Limitations

This study is subject to several limitations warranting consideration. Firstly, the sample sizes of post-stroke patients in both cross-sectional and longitudinal experiments were relatively small. Although our results displayed a large effect size and were validated by two distinct methods, a large-sample study needs to verify our findings in the future. Secondly, as all stroke patients exhibit unilateral subcortical lesions, and therefore, the observed patterns of functional reorganization may not necessarily be generalized to patients with other types of brain lesions. Thirdly, there was a pronounced male predominance in the present study, which may be due to the protective role of female hormones for stroke incidence and the increased vulnerability of males to stroke [57]. Despite controlling for gender as a nuisance covariate in our statistical analyses, future studies need to maintain an appropriate gender balance to confirm our findings. Fourth, we utilized a small-sample longitudinal dataset to confirm the relationships revealed by the cross-sectional dataset. However, further validation of FCD's predictive efficacy for motor outcomes post-stroke requires a large-sample longitudinal study in the future. Last but not least, the cross-sectional design employed in this study did not allow for a conclusion to be drawn on the causes of the functional changes in the left inferior frontal gyrus. Therefore, how such changes contribute to motor dysfunction remains speculative. Future studies adopting a prospective design on a larger sample size and multiple brain lesion groups can facilitate a better understanding of the mechanisms underlying motor dysfunction in post-stroke patients without aphasia.

## Conclusions

The current study explored functional alterations in non-acute stroke patients following left and right hemisphere subcortical lesions, using the distance-dependent FCD approach. Our results demonstrated that the left inferior frontal gyrus exhibited distance-independent FCD changes, while the bilateral supplementary motor gyrus, cerebellum anterior/posterior lobe, and left middle occipital gyrus showed distance-related FCD alterations in stroke patients, regardless of the lesion side. Importantly, our study highlights that these changes in FCD have the potential to predict clinical function, with the correlations between FCD values in the left inferior frontal gyrus and bilateral supplementary motor gyrus and specific paralyzed motor function in all non-acute stroke patients without aphasia. These findings offer additional evidence to comprehend the neurophysiological mechanisms underlying motor dysfunctions following non-acute stroke.

### Supplementary Information

Supplementary Material 1: Fig. S1. Lesion display for each patient. The red region represents individual lesion. L, left; R, right; LSS, left subcortical stroke; RSS, right subcortical stroke. Fig. S2. Comparison of mean gFCD, lFCD, and sFCD among the LSS, RSS, and HCs. LSS, left subcortical stroke; RSS, right subcortical stroke; HCs, healthy controls; FCD, functional connectivity density; gFCD, global functional connectivity density; lFCD, long-range functional connectivity density; sFCD, short-range functional connectivity density. *, 0.01 < *P* < 0.05; **, 0.001 < *P* < 0.01; ***, *P* < 0.001; ns, not significant. Fig. S3. Leave-one-out cross-validation analysis result for the ANCOVA among LSS, RSS, and HCs. LSS, left subcortical stroke; RSS, right subcortical stroke; HCs, healthy controls; gFCD, global functional connectivity density; lFCD, long-range functional connectivity density; sFCD, short-range functional connectivity density; SMA, supplementary motor area; IFG, inferior frontal gyrus; CPL, cerebellum posterior lobe; MOG, middle occipital gyrus; CAL, cerebellum anterior lobe. Fig. S4. Alterations in FC with the left IFG as the seed in stroke patients. A and B represent the results of ANCOVA and post-hoc test, respectively. IFG, inferior frontal gyrus; PreCG, precentral gyrus; PosCG, postcentral gyrus; MTG, middle temporal gyrus; MFG, middle frontal gyrus; LSS, left subcortical stroke; RSS, right subcortical stroke; HCs, healthy controls. *, 0.01 < *P* < 0.05; **, 0.001 < *P* < 0.01; ***, *P* < 0.001; ns, not significant.

## Data Availability

The datasets used and/or analyzed during the current study are available from the corresponding author on reasonable request.

## Abbreviations

ANCOVA: One-way analysis of covariance
FC: Functional connectivity
FCD: Functional connectivity density
FMA: Fugl-Meyer Assessment
FMA-LE: The Fugl-Meyer Assessment Lower Extremity
FMA-UE: The Fugl-Meyer Assessment Upper Extremity
gFCD: Global-range functional connectivity density
HCs: Healthy controls
lFCD: Long-range functional connectivity density
LSS: Left subcortical stroke
MBI-C: Chinese version of Modified Barthel Index
RSS: Right subcortical stroke
sFCD: Short-range functional connectivity density

## References

[1] Carter AR, Shulman GL, Corbetta M (2012). Why use a connectivity-based approach to study stroke and recovery of function?. Neuroimage.

[2] Liu F, Chen C, Hong W, Bai Z, Wang S, Lu H, Lin Q, Zhao Z, Tang C (2022). Selectively disrupted sensorimotor circuits in chronic stroke with hand dysfunction. CNS Neurosci Ther.

[3] Zhao Y, Cox CR, Lambon Ralph MA, Halai AD (2023). Using in vivo functional and structural connectivity to predict chronic stroke aphasia deficits. Brain.

[4] Zhao Z, Cai H, Huang M, Zheng W, Liu T, Sun D, Han G, Ni L, Zhang Y, Wu D (2021). Altered Functional connectivity of hippocampal subfields in poststroke dementia. J Magn Reson Imaging.

[5] Tang C, Zhao Z, Chen C, Zheng X, Sun F, Zhang X, Tian J, Fan M, Wu Y, Jia J (2016). Decreased functional connectivity of homotopic brain regions in chronic stroke patients: a resting state fMRI study. PLoS One.

[6] Zhao Z, Wang X, Fan M, Yin D, Sun L, Jia J, Tang C, Zheng X, Jiang Y, Wu J (2016). Altered effective connectivity of the primary motor cortex in stroke: a resting-state fMRI study with granger causality analysis. PLoS One.

[7] Zuo XN, Ehmke R, Mennes M, Imperati D, Castellanos FX, Sporns O, Milham MP (2012). Network centrality in the human functional connectome. Cereb Cortex.

[8] Shan A, Zhang H, Gao M, Wang L, Cao X, Gan C, Sun H, Yuan Y, Zhang K (2023). Aberrant voxel-based degree centrality and functional connectivity in Parkinson's disease patients with fatigue. CNS Neurosci Ther.

[9] Min Y, Liu C, Zuo L, Wang Y, Li Z (2023). The relationship between altered degree centrality and cognitive function in mild subcortical stroke: a resting-state fMRI study. Brain Res.

[10] Yao G, Li J, Liu S, Wang J, Cao X, Li X, Cheng L, Chen H, Xu Y (2020). Alterations of functional connectivity in stroke patients with basal ganglia damage and cognitive impairment. Front Neurol.

[11] Zhang S, Li B, Liu K, Hou X, Zhang P (2022). Abnormal voxel-based degree centrality in patients with postpartum depression: a resting-state functional magnetic resonance imaging study. Front Neurosci.

[12] Liao H, Yi J, Cai S, Shen Q, Liu Q, Zhang L, Li J, Mao Z, Wang T, Zi Y (2021). Changes in degree centrality of network nodes in different frequency bands in Parkinson's disease with depression and without depression. Front Neurosci.

[13] Liang X, Zou Q, He Y, Yang Y (2013). Coupling of functional connectivity and regional cerebral blood flow reveals a physiological basis for network hubs of the human brain. Proc Natl Acad Sci U S A.

[14] Achard S, Salvador R, Whitcher B, Suckling J, Bullmore E (2006). A resilient, low-frequency, small-world human brain functional network with highly connected association cortical hubs. J Neurosci.

[15] Sheng J, Zhang L, Feng J, Liu J, Li A, Chen W, Shen Y, Wang J, He Y, Xue G (2021). The coupling of BOLD signal variability and degree centrality underlies cognitive functions and psychiatric diseases. Neuroimage.

[16] Tomasi D, Volkow ND (2012). Laterality patterns of brain functional connectivity: gender effects. Cereb Cortex.

[17] Chen X, Zhang Z, Zhang Q, Zhao W, Zhai J, Chen M, Du B, Deng X, Ji F, Wang C (2018). Effect of rs1344706 in the ZNF804A gene on the brain network. Neuroimage Clin.

[18] Chen S, Qian A, Tao J, Zhou R, Fu C, Yang C, Lin Q, Zhou J, Li J, Huang X (2022). Different effects of the DRD4 genotype on intrinsic brain network connectivity strength in drug-naïve children with ADHD and healthy controls. Brain Imaging Behav.

[19] Yang Y, Cui Q, Pang Y, Chen Y, Tang Q, Guo X, Han S, Ameen Fateh A, Lu F, He Z (2021). Frequency-specific alteration of functional connectivity density in bipolar disorder depression. Prog Neuropsychopharmacol Biol Psychiatry.

[20] Wang J, Wei Q, Yuan X, Jiang X, Xu J, Zhou X, Tian Y, Wang K (2018). Local functional connectivity density is closely associated with the response of electroconvulsive therapy in major depressive disorder. J Affect Disord.

[21] Hong W, Lin Q, Cui Z, Liu F, Xu R, Tang C (2019). Diverse functional connectivity patterns of resting-state brain networks associated with good and poor hand outcomes following stroke. Neuroimage Clin.

[22] Wu W, Zhang Y, Jiang J, Lucas MV, Fonzo GA, Rolle CE, Cooper C, Chin-Fatt C, Krepel N, Cornelssen CA (2020). An electroencephalographic signature predicts antidepressant response in major depression. Nat Biotechnol.

[23] Liu X, Qiu S, Wang X, Chen H, Tang Y, Qin Y (2023). Aberrant dynamic functional-structural connectivity coupling of large-scale brain networks in poststroke motor dysfunction. Neuroimage Clin.

[24] Goodin P, Lamp G, Vidyasagar R, McArdle D, Seitz RJ, Carey LM (2018). Altered functional connectivity differs in stroke survivors with impaired touch sensation following left and right hemisphere lesions. Neuroimage Clin.

[25] Faul F, Erdfelder E, Buchner A, Lang AG (2009). Statistical power analyses using G*Power 3.1: tests for correlation and regression analyses. Behav Res Methods.

[26] Arya KN, Verma R, Garg RK (2011). Estimating the minimal clinically important difference of an upper extremity recovery measure in subacute stroke patients. Top Stroke Rehabil.

[27] Kwong PWH, Ng SSM (2019). Cutoff score of the lower-extremity motor subscale of Fugl-Meyer assessment in chronic stroke survivors: a cross-sectional study. Arch Phys Med Rehabil.

[28] Leung SO, Chan CC, Shah S (2007). Development of a Chinese version of the Modified Barthel Index– validity and reliability. Clin Rehabil.

[29] Yan CG, Wang XD, Zuo XN, Zang YF (2016). DPABI: data processing & analysis for (resting-state) brain imaging. Neuroinformatics.

[30] Friston KJ, Williams S, Howard R, Frackowiak RS, Turner R (1996). Movement-related effects in fMRI time-series. Magn Reson Med.

[31] Beucke JC, Sepulcre J, Talukdar T, Linnman C, Zschenderlein K, Endrass T, Kaufmann C, Kathmann N (2013). Abnormally high degree connectivity of the orbitofrontal cortex in obsessive-compulsive disorder. JAMA Psychiatry.

[32] Sonoda T, Matsuzaki J, Yamamoto Y, Sakurai T, Aoki Y, Takizawa S, Niida S, Ochiya T (2019). Serum MicroRNA-based risk prediction for stroke. Stroke.

[33] Jacobs JV, Lou JS, Kraakevik JA, Horak FB (2009). The supplementary motor area contributes to the timing of the anticipatory postural adjustment during step initiation in participants with and without Parkinson's disease. Neuroscience.

[34] Yin D, Song F, Xu D, Sun L, Men W, Zang L, Yan X, Fan M (2014). Altered topological properties of the cortical motor-related network in patients with subcortical stroke revealed by graph theoretical analysis. Hum Brain Mapp.

[35] Yin D, Luo Y, Song F, Xu D, Peterson BS, Sun L, Men W, Yan X, Fan M (2013). Functional reorganization associated with outcome in hand function after stroke revealed by regional homogeneity. Neuroradiology.

[36] Chen P, Hu R, Gao L, Wu B, Peng M, Jiang Q, Wu X, Xu H (2021). Abnormal degree centrality in end-stage renal disease (ESRD) patients with cognitive impairment: a resting-state functional MRI study. Brain Imaging Behav.

[37] Jiang C, Yi L, Cai S, Zhang L (2019). Ischemic stroke in pontine and corona radiata: location specific impairment of neural network investigated with resting state fMRI. Front Neurol.

[38] Yin D, Song F, Xu D, Peterson BS, Sun L, Men W, Yan X, Fan M (2012). Patterns in cortical connectivity for determining outcomes in hand function after subcortical stroke. PLoS One.

[39] Shamay-Tsoory SG, Aharon-Peretz J, Perry D (2009). Two systems for empathy: a double dissociation between emotional and cognitive empathy in inferior frontal gyrus versus ventromedial prefrontal lesions. Brain.

[40] Wang J, Yang Y, Zhao X, Zuo Z, Tan LH (2020). Evolutional and developmental anatomical architecture of the left inferior frontal gyrus. Neuroimage.

[41] Rizzolatti G, Craighero L (2004). The mirror-neuron system. Annu Rev Neurosci.

[42] Rizzolatti G, Luppino G (2001). The cortical motor system. Neuron.

[43] Buccino G, Vogt S, Ritzl A, Fink GR, Zilles K, Freund HJ, Rizzolatti G (2004). Neural circuits underlying imitation learning of hand actions: an event-related fMRI study. Neuron.

[44] Wang X, Wang H, Xiong X, Sun C, Zhu B, Xu Y, Fan M, Tong S, Sun L, Guo X (2020). Motor imagery training after stroke increases slow-5 oscillations and functional connectivity in the ipsilesional inferior parietal lobule. Neurorehabil Neural Repair.

[45] Garrison KA, Aziz-Zadeh L, Wong SW, Liew SL, Winstein CJ (2013). Modulating the motor system by action observation after stroke. Stroke.

[46] Ma ZZ, Wu JJ, Hua XY, Zheng MX, Xing XX, Ma J, Li SS, Shan CL, Xu JG (2022). Brain function and upper limb deficit in stroke with motor execution and imagery: a cross-sectional functional magnetic resonance imaging study. Front Neurosci.

[47] Larivière S, Ward NS, Boudrias MH (2018). Disrupted functional network integrity and flexibility after stroke: relation to motor impairments. Neuroimage Clin.

[48] Gan C, Wang M, Si Q, Yuan Y, Zhi Y, Wang L, Ma K, Zhang K (2020). Altered interhemispheric synchrony in Parkinson's disease patients with levodopa-induced dyskinesias. NPJ Parkinsons Dis.

[49] Garcea FE, Stoll H, Buxbaum LJ (2019). Reduced competition between tool action neighbors in left hemisphere stroke. Cortex.

[50] Chen J, Sun D, Shi Y, Jin W, Wang Y, Xi Q, Ren C (2018). Dynamic alterations in spontaneous neural activity in multiple brain networks in subacute stroke patients: a resting-state fMRI study. Front Neurosci.

[51] Almeida SRM, Stefano Filho CA, Vicentini J, Novi SL, Mesquita RC, Castellano G, Li LM (2022). Modeling functional network topology following stroke through graph theory: functional reorganization and motor recovery prediction. Braz J Med Biol Res.

[52] Liang L, Hu R, Luo X, Feng B, Long W, Song R (2020). Reduced complexity in stroke with motor deficits: a resting-state fMRI study. Neuroscience.

[53] Hanakawa T, Hotta F, Nakamura T, Shindo K, Ushiba N, Hirosawa M, Yamazaki Y, Moriyama Y, Takagi S, Mizuno K (2023). Macrostructural cerebellar neuroplasticity correlates with motor recovery after stroke. Neurorehabil Neural Repair.

[54] Xu X, Jang I, Zhang J, Zhang M, Wang L, Ye G, Zhao A, Zhang Y, Li B, Liu J (2024). Cortical gray to white matter signal intensity ratio as a sign of neurodegeneration and cognition independent of β-amyloid in dementia. Hum Brain Mapp.

[55] Fujimoto H, Mihara M, Hattori N, Hatakenaka M, Kawano T, Yagura H, Miyai I, Mochizuki H (2014). Cortical changes underlying balance recovery in patients with hemiplegic stroke. Neuroimage.

[56] Mihara M, Fujimoto H, Hattori N, Otomune H, Kajiyama Y, Konaka K, Watanabe Y, Hiramatsu Y, Sunada Y, Miyai I (2021). Effect of neurofeedback facilitation on poststroke gait and balance recovery: a randomized controlled trial. Neurology.

[57] Gibson CL, Attwood L (2016). The impact of gender on stroke pathology and treatment. Neurosci Biobehav Rev.

