# Supplementary material for: Distance-related functional reorganization predicts motor outcome in stroke patients
Source: BMC Med. 2024 Jun 18;22:247. doi: 10.1186/s12916-024-03435-7 (PMC11184708; doi:10.1186/s12916-024-03435-7)
Supplement: Supplementary file 1 — Supplementary Material 1: Fig. S1. Lesion display for each patient. The red region represents individual lesion. L, left; R, right; LSS, left subcortical stroke; RSS, right subcortical stroke. Fig. S2. Comparison of mean gFCD, lFCD, and sFCD among the LSS, RSS, and HCs. LSS, left subcortical stroke; RSS, right subcortical stroke; HCs, healthy controls; FCD, functional connectivity density; gFCD, global functional connectivity density; lFCD, long-range functional connectivity density; sFCD, short-range functional connectivity density. *, 0.01 < P < 0.05; **, 0.001 < P < 0.01; ***, P < 0.001; ns, not significant. Fig. S3. Leave-one-out cross-validation analysis result for the ANCOVA among LSS, RSS, and HCs. LSS, left subcortical stroke; RSS, right subcortical stroke; HCs, healthy controls; gFCD, global functional connectivity density; lFCD, long-range functional connectivity density; sFCD, short-range functional connectivity density; SMA, supplementary motor area; IFG, inferior frontal gyrus; CPL, cerebellum posterior lobe; MOG, middle occipital gyrus; CAL, cerebellum anterior lobe. Fig. S4. Alterations in FC with the left IFG as the seed in stroke patients. A and B represent the results of ANCOVA and post-hoc test, respectively. IFG, inferior frontal gyrus; PreCG, precentral gyrus; PosCG, postcentral gyrus; MTG, middle temporal gyrus; MFG, middle frontal gyrus; LSS, left subcortical stroke; RSS, right subcortical stroke; HCs, healthy controls. *, 0.01 < P < 0.05; **, 0.001 < P < 0.01; ***, P < 0.001; ns, not significant. [file 12916_2024_3435_MOESM1_ESM.docx]

**Additional Methods**

**Functional connectivity analysis with the left inferior frontal gyrus as the seed**

The current study observed that the alterations of functional connectivity density (FCD) in the left inferior frontal gyrus were distance- and lesion-independent, encompassing abnormal changes in global, long-range, and short-range FCD in both left subcortical stroke (LSS) and right subcortical stroke (RSS) patients compared to the healthy controls (HCs) group. This finding was interesting in non-acute stroke patients with motor impairment but no aphasia, prompting us to further analyze functional connectivity (FC) with the left inferior frontal gyrus as the seed region. The specific steps are as follows: First, the voxel-wise FC analysis was performed on the resting-state data of all subjects within the region of interest. In the next step, the blood oxygen level-dependent time series of voxels within the seed region were averaged to obtain a reference time series. For each subject and seed region, a correlogram was generated by calculating the correlation coefficient between the reference time series and the time series of the remaining whole-brain voxels. Finally, the correlation coefficients were converted to *z*-values ​​using Fisher *r*-to-*z* transformation to improve normality.

**Additional Results**


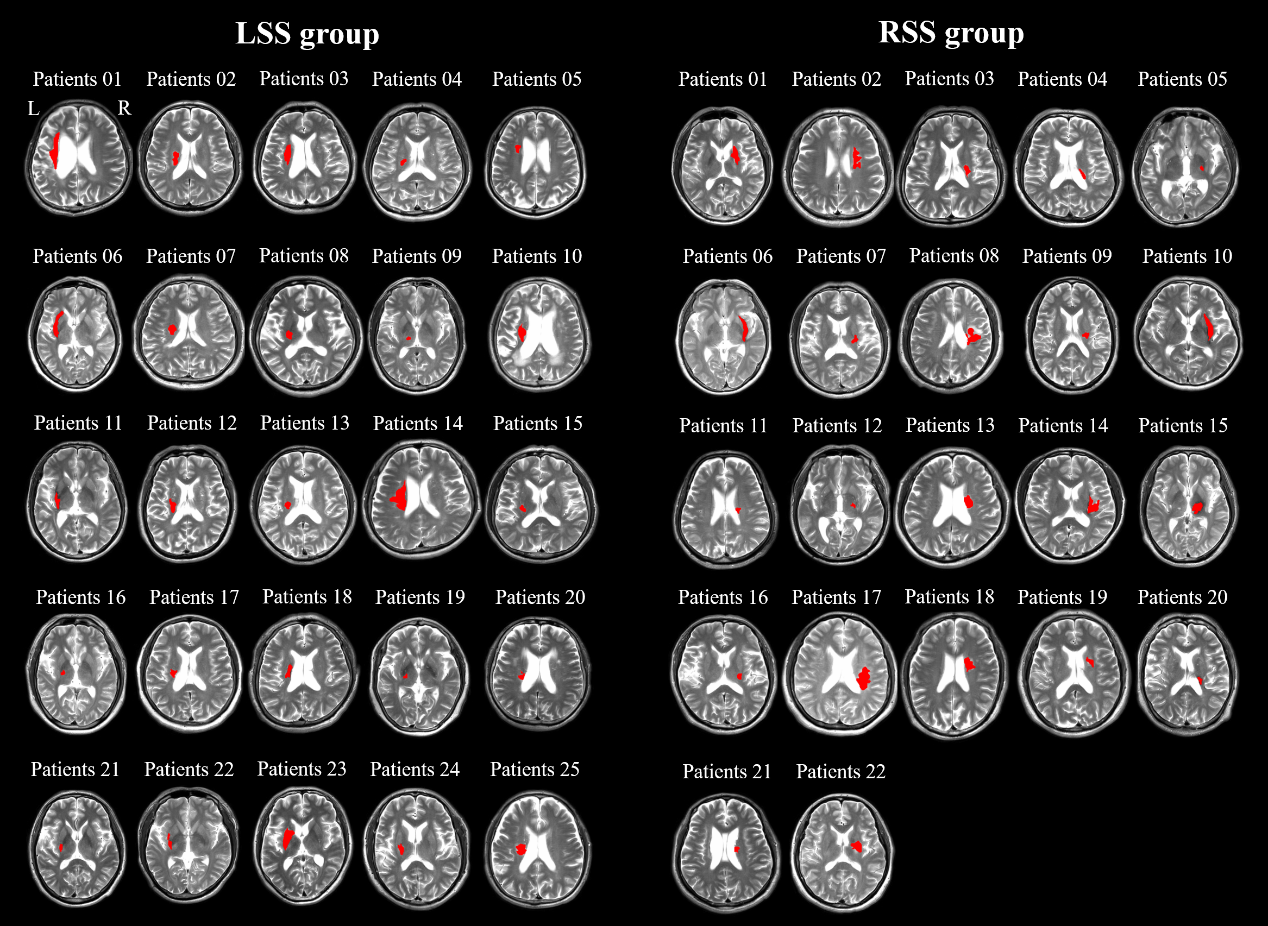


**Fig. S1. Lesion display for each patient.** The red region represents individual lesion. L, left; R, right; LSS, left subcortical stroke; RSS, right subcortical stroke.


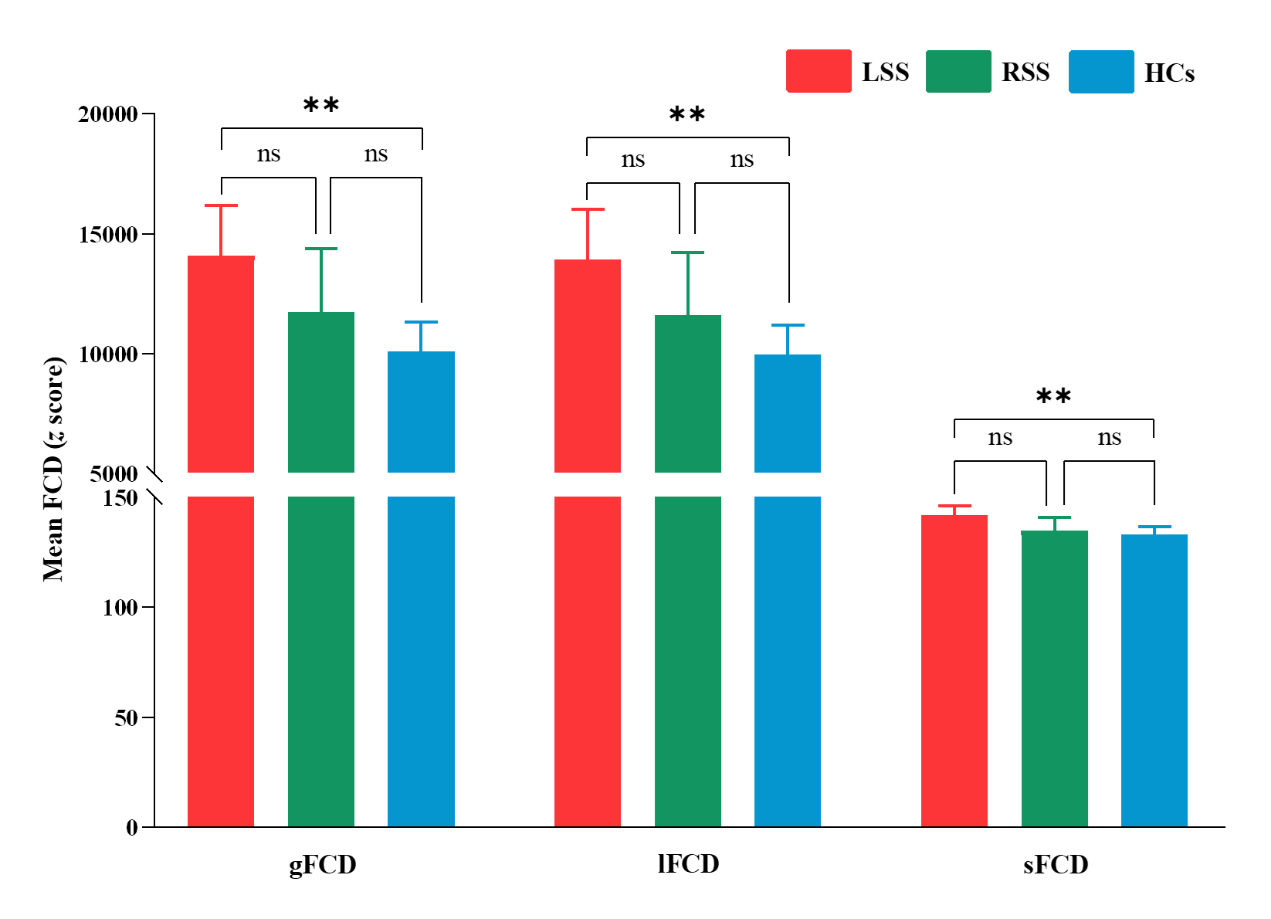


**Fig. S2. Comparison of mean gFCD, lFCD, and sFCD among the LSS, RSS, and HCs.** LSS, left subcortical stroke; RSS, right subcortical stroke; HCs, healthy controls; FCD, functional connectivity density; gFCD, global functional connectivity density; lFCD, long-range functional connectivity density; sFCD, short-range functional connectivity density. *, 0.01< *P* < 0.05; **, 0.001< *P* < 0.01; ***, *P* < 0.001; ns, not significant.


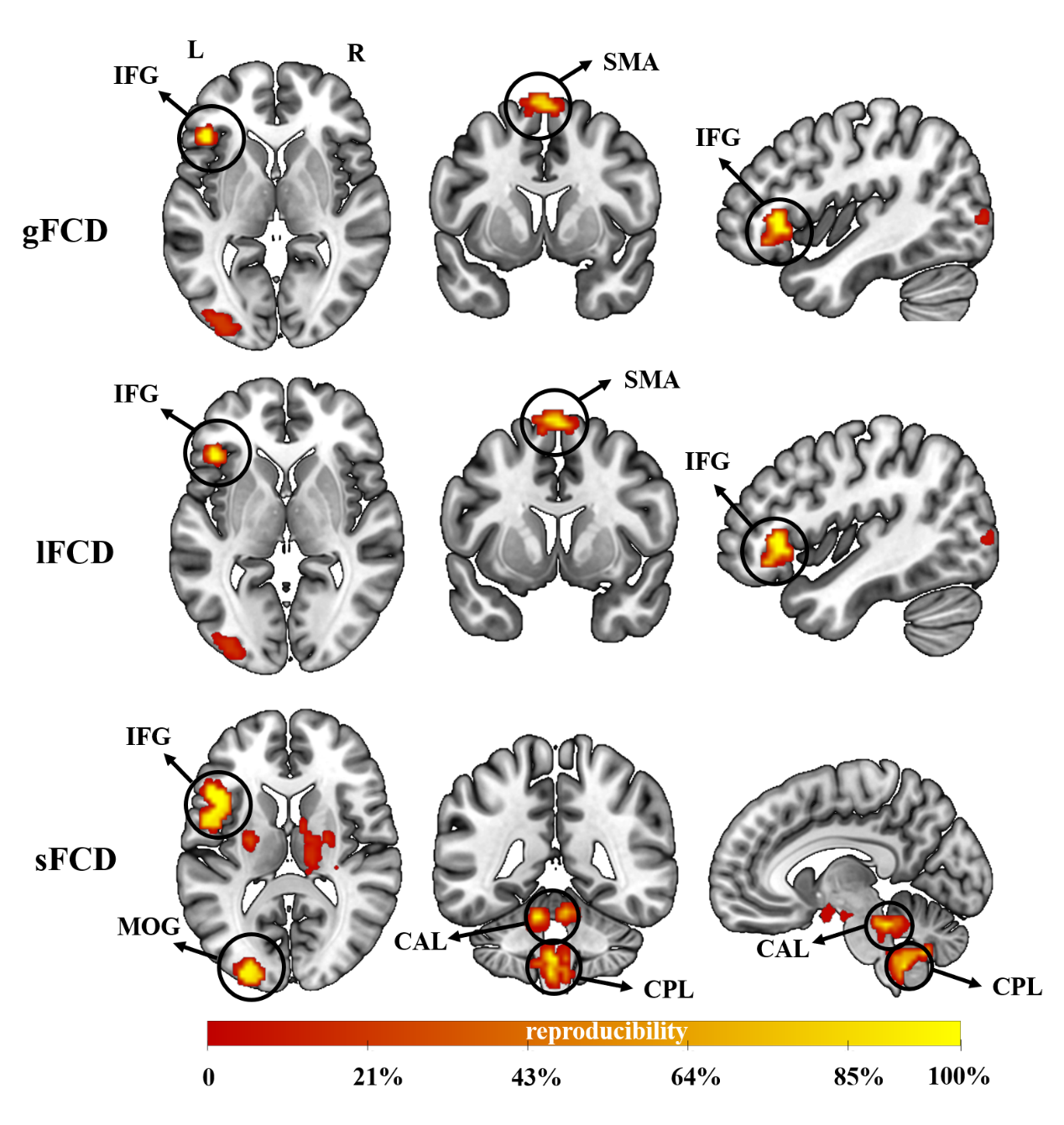


**Fig. S3. Leave-one-out cross-validation analysis result for the ANCOVA among LSS, RSS, and HCs.** LSS, left subcortical stroke; RSS, right subcortical stroke; HCs, healthy controls; gFCD, global functional connectivity density; lFCD, long-range functional connectivity density; sFCD, short-range functional connectivity density; SMA, supplementary motor area; IFG, inferior frontal gyrus; CPL, cerebellum posterior lobe; MOG, middle occipital gyrus; CAL, cerebellum anterior lobe.

The analysis of covariance (ANCOVA) showed significant differences in FC between the left inferior frontal gyrus and the bilateral middle temporal gyrus, right precentral gyrus, right postcentral gyrus, right middle occipital gyrus, left precuneus, and right middle frontal gyrus (Additional file 1: **Table S3** and **Fig. S4A**). Subsequent *post-hoc* comparisons exhibited that both the LSS and RSS groups experienced a significant increase in these FCs compared to the HCs (Additional file 1: **Table S3** and **Fig. S4B**, |Cohen's *d|>*0.8). However, no significant differences in the FC values were observed between the LSS and RSS groups (Additional file 1: **Table S3**).


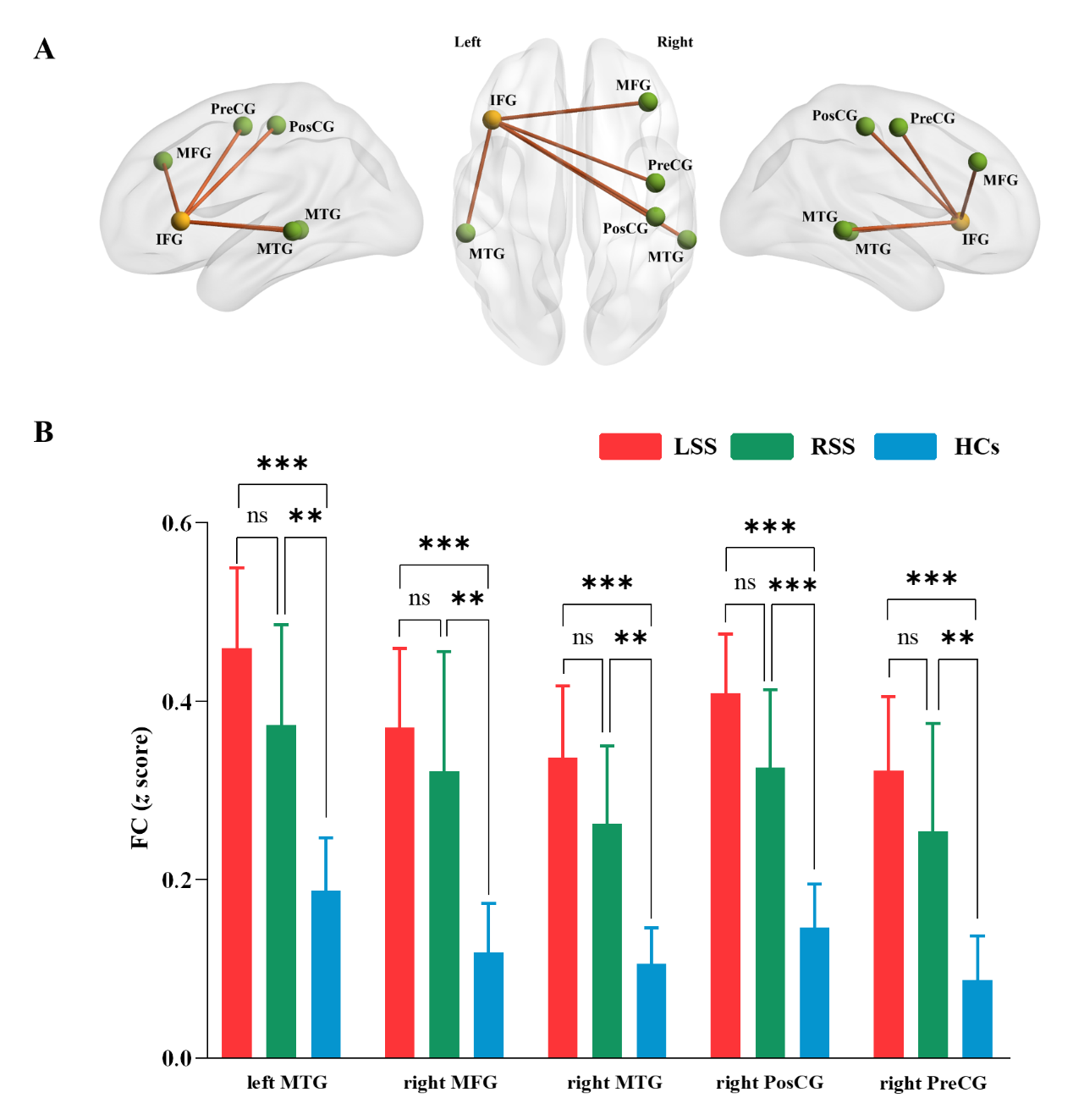


**Fig. S4. Alterations in FC with the left IFG as the seed in stroke patients.** A and B represent the results of ANCOVA and post-hoc test, respectively. IFG, inferior frontal gyrus; PreCG, precentral gyrus; PosCG, postcentral gyrus; MTG, middle temporal gyrus; MFG, middle frontal gyrus; LSS, left subcortical stroke; RSS, right subcortical stroke; HCs, healthy controls. *, 0.01< *P* < 0.05; **, 0.001< *P* < 0.01; ***, *P* < 0.001; ns, not significant.

**Table S1.** Regions showing significant differences in gFCD, lFCD, and sFCD among LSS, RSS, and HCs, using a distance criterion of 6 mm.

| Regions | Hemisphere | MNI coordinates | | | Cluster size | *F* value | LSS vs. HCs | | RSS vs. HCs | | LSS vs. RSS | |
| --- | --- | --- | --- | --- | --- | --- | --- | --- | --- | --- | --- | --- |
|  |  | x | y | z |  |  | *t* value | Cohen’ *d* | *t* value | Cohen’ *d* | *t* value | Cohen’ *d* |
| *gFCD* | | | | | | | | | | | | |
| Inferior frontal gyrus | Left | -45 | 27 | 3 | 39 | 11.29 | 4.99*** | 1.23 | 4.54*** | 1.17 | 0.50 | 0.15 |
| Supplementary motor area | Bilateral | 9 | 3 | 72 | 23 | 9.29 | 3.96*** | 1.02 | 4.24*** | 1.15 | -0.32 | -0.09 |
| *lFCD* | | | | | | | | | | | | |
| Inferior frontal gyrus | Left | -45 | 27 | 3 | 39 | 11.29 | 4.99*** | 1.23 | 4.54*** | 1.17 | 0.50 | 0.15 |
| Supplementary motor area | Bilateral | 9 | 3 | 72 | 23 | 9.29 | 3.96*** | 1.02 | 4.24*** | 1.15 | -0.32 | -0.09 |
| *sFCD* | | | | | | | | | | | | |
| Cerebellum posterior lobe | Bilateral | 0 | -54 | -48 | 49 | 12.82 | -4.52*** | -1.18 | -3.77*** | -1.02 | -0.47 | -0.14 |
| Cerebellum posterior lobe | Left | -27 | -63 | -39 | 36 | 8.67 | -3.69*** | -0.96 | -2.94*** | -0.80 | -0.62 | -0.18 |
| Inferior frontal gyrus | Left | -45 | 15 | 12 | 36 | 13.24 | 8.44*** | 2.03 | 5.53*** | 1.53 | 1.45 | 0.43 |
| Precentral gyrus | Left |  |  |  | 11 |  |  |  |  |  |  |  |

Note: MNI, Montreal Neurological Institute; LSS, left subcortical stroke; RSS, right subcortical stroke; HCs, healthy controls; gFCD, global functional connectivity density; lFCD, long-range functional connectivity density; sFCD, short-range functional connectivity density. *, 0.01< *P* < 0.05; **, 0.001< *P* < 0.01; ***, *P* < 0.001.

**Table S2.** Regions showing significant differences in gFCD, lFCD, and sFCD among LSS, RSS, and HCs, using a distance criterion of 18mm.

| Regions | Hemisphere | MNI coordinates | | | Cluster size | *F* value | LSS vs. HCs | | RSS vs. HCs | | LSS vs. RSS | |
| --- | --- | --- | --- | --- | --- | --- | --- | --- | --- | --- | --- | --- |
|  |  | x | y | z |  |  | *t* value | Cohen’ *d* | *t* value | Cohen’ *d* | *t* value | Cohen’ *d* |
| *gFCD* | | | | | | | | | | | | |
| Inferior frontal gyrus | Left | -45 | 27 | 3 | 39 | 11.29 | 4.61*** | 1.23 | 4.54*** | 1.17 | 0.50 | 0.15 |
| Supplementary motor area | Bilateral | 9 | 3 | 72 | 23 | 9.29 | 3.96*** | 1.02 | 4.24*** | 1.15 | -0.32 | -0.09 |
| *lFCD* | | | | | | | | | | | | |
| Inferior frontal gyrus | Left | -45 | 27 | 3 | 39 | 11.27 | 4.63*** | 1.23 | 4.50*** | 1.17 | 0.52 | 0.15 |
| Supplementary motor area | Bilateral | 9 | 3 | 72 | 22 | 9.24 | 3.97*** | 1.02 | 4.23*** | 1.15 | -0.31 | -0.09 |
| *sFCD* | | | | | | | | | | | | |
| Cerebellum posterior lobe | Bilateral | 0 | -54 | -51 | 51 | 11.92 | -3.13*** | -0.83 | -4.11*** | -1.01 | 0.53 | 0.16 |
| Cerebellum anterior lobe | Bilateral | 0 | -33 | -21 | 25 | 10.00 | -4.17*** | -1.03 | -3.30** | -0.82 | -1.11 | -0.33 |
| Middle occipital gyrus | Left | -24 | -99 | 12 | 87 | 10.05 | -3.50** | -0.92 | -3.98*** | -1.07 | 0.80 | 0.23 |
| Inferior frontal gyrus | Left | -42 | 12 | 12 | 73 | 12.40 | 5.25*** | 1.32 | 3.93*** | 1.02 | 0.87 | 0.25 |
| Precentral gyrus | Left |  |  |  | 26 |  |  |  |  |  |  |  |

Note: MNI, Montreal Neurological Institute; LSS, left subcortical stroke; RSS, right subcortical stroke; HCs, healthy controls; gFCD, global functional connectivity density; lFCD, long-range functional connectivity density; sFCD, short-range functional connectivity density. *,0.01< *P* < 0.05; **, 0.001< *P* < 0.01; ***, *P* < 0.001.

**Table S3.** Regions showing significant differences in functional connectivity of left inferior frontal gyrus among LSS, RSS, and HCs.

| Regions | Hemisphere | MNI coordinates | | | Cluster size | *F* value | LSS vs. HCs | | RSS vs. HCs | | LSS vs. RSS | |
| --- | --- | --- | --- | --- | --- | --- | --- | --- | --- | --- | --- | --- |
|  |  | x | y | z |  |  | *t* value | Cohen’ *d* | *t* value | Cohen’ *d* | *t* value | Cohen’ *d* |
| Middle temporal gyrus | Left | -63 | -6 | -6 | 68 | 13.57 | 5.39*** | 1.36 | 3.31** | 0.84 | 1.26 | 0.36 |
| Middle frontal gyrus | Right | 45 | 6 | 42 | 44 | 14.67 | 5.21*** | 1.31 | 2.91** | 0.82 | 0.65 | 0.19 |
| Middle temporal gyrus | Right | 60 | 0 | -9 | 75 | 14.76 | 5.29*** | 1.42 | 3.37** | 0.95 | 1.30 | 0.38 |
| Postcentral gyrus | Right | 3 | -39 | 78 | 250 | 17.19 | 6.63*** | 1.69 | 4.00*** | 1.03 | 1.59 | 0.46 |
| Middle frontal gyrus | Right |  |  |  | 233 |  |  |  |  |  |  |  |
| Precuneus | Left |  |  |  | 103 |  |  |  |  |  |  |  |
| Precentral gyrus | Right | 66 | -6 | 12 | 40 | 13.54 | 5.28*** | 1.31 | 3.08** | 0.75 | 0.98 | 0.28 |

Note: MNI, Montreal Neurological Institute; LSS, left subcortical stroke; RSS, right subcortical stroke; HCs, healthy controls. *, 0.01< *P* < 0.05; **, 0.001< *P* < 0.01; ***, *P* < 0.001.
